# Microbubble-mediated ultrasound promotes accumulation of bone marrow mesenchymal stem cell to the prostate for treating chronic bacterial prostatitis in rats

**DOI:** 10.1038/srep19745

**Published:** 2016-01-22

**Authors:** Shanhong Yi, Guangwei Han, Yonggang Shang, Chengcheng Liu, Dong Cui, Shuangjiang Yu, Bin Liao, Xiang Ao, Guangzhi Li, Longkun Li

**Affiliations:** 1 Department of Urology, Xinqiao Hospital, Third Military Medical University, Chongqing, 400037, China; 2 Department of Urology, The 169th hospital of People’s Liberation Army, Hengyang, Hunan Province, 421002, China; 3 Department of Urology, Chongqing Emergency Medical Center, Chongqing, 400014, China

## Abstract

Chronic bacterial prostatitis (CBP) is an intractable disease. Although bone marrow mesenchymal stem cells (BMMSCs) are able to regulate inflammation in CBP, the effect of microbubble-mediated ultrasound- induced accumulation of BMMSCs on CBP remains unclear. To address this gap, a model of CBP was established in SD rats, which were then treated with BMMSCs alone (BMMSC group), BMMSCs with ultrasound (ultrasound group), BMMSCs with microbubble-mediated ultrasound (MMUS group) and compared with a healthy control group. A therapeutic-ultrasound apparatus was used to treat the prostate in the presence of circulating microbubbles and BMMSCs. The BMMSC distribution was assessed with *in vivo* imaging, and the prostate structure with light microscopy. Real-time quantitative RT-PCR, ELISA, and immunohistochemistry were used to assess the expressions of TNF-α and IL-1β. More BMMSCs were found in the prostate in the MMUS group than in the CBP, ultrasound, and BMMSC groups. Inflammatory cell infiltration, fibrous tissue hyperplasia, and tumor-like epithelial proliferation were significantly reduced in the MMUS group, as were the mRNA and protein expressions of TNF-α and IL-1β. Microbubble-mediated ultrasound-induced accumulation of BMMSCs can inhibit inflammation and decrease TNF-α and IL-1β expressions in the prostate of CBP rats, suggesting that this method may be therapeutic for CPB.

Chronic prostatitis (CP) is a common disease in adult males^1^, with a prevalence of 5–14%^2^. In order to better diagnose and treat this disease, the National Institutes of Health divided prostatitis into four types: I. acute bacterial prostatitis (APB); II. chronic bacterial prostatitis (CBP); III. chronic prostatitis/chronic pelvic pain syndrome(CP/CPPS); and IV. asymptomatic inflammatory prostatitis^3^. The prevalence of one type of CP, CBP, accounts for about 10% of all CP patients^4^. CBP which is caused by bacterial infection maintains irritative urinary symptoms, pelvic discomfort, and pain during or following ejaculation^5^. Although bacterial infection is understood as the primary cause, the best therapeutic treatment for CBP remains unclear.

Many drugs cannot fully enter the prostate tissues^6^. The composition and structure of the prostate suggest it acts as a “blood-prostate barrier”^7^, which similar to the blood-brain barrier, displays permselectivity against many macromolecules. The blood-prostate barrier is the main impediment to pharmaceutical CBP treatment^8^. Therefore, many recent research efforts have attempted to bypass this barrier to improve the permeability of the prostate. Previous study has showed that microbubble-mediated ultrasound can open the blood-brain barrier and increase the concentrations of drugs in the brain^9^. Similarly, two studies suggested that the permeability of prostate tissue was significantly increased after microbubble-mediated ultrasound irradiation^10^,^11^, suggesting that microbubble-mediated ultrasound may be an effective treatment method for CBP.

In a previous study from our group^12^, we found no bacterial growth after treatment with antibiotics for 2 weeks in an ABP rat model. However, the inflammatory reaction continued and many of the inflammatory cytokines were not decreased after treatment. These results indicated that inflammatory cytokines play important roles in the development of CBP and that antibiotics can inhibit bacterial growth, but cannot prevent the development of inflammation.

Bone marrow mesenchymal stem cells (BMMSCs) are a cell population characterized by rapid proliferation, low immunogenicity, pluripotency, tissue repair ability, and immunosuppression^13^,^14^. BMMSCs have been used in the treatment of many diseases, such as graft-versus-host disease^15^ and Crohn’s disease^16^. BMMSCs can suppress the immune-mediated inflammation by inhibiting T cells, B cells, antigen presenting cells, and the production of pro-inflammatory cytokines. In one study where rats with bronchial asthma were given an intravenous injection of BMMSCs, after 1 hour, the cells had gathered in the sites of inflammation in the lung and reduced the inflammation^17^. Similarly, *E. coli*-induced pneumonia was clearly reduced after injecting BMMSCs into the trachea^18^. These studies show that BMMSCs can play a positive role in the treatment of different inflammatory diseases. Whether BMMSCs reduce the intractable inflammatory state of CBP remains an open question because few studies have been reported on this topic. Thus, the aim of the present study was to assess the therapeutic effects of microbubble-mediated ultrasound-induced accumulation of BMMSCs on CBP in a rat model.

## Methods

### Animals

The methods of our study were performed out accordance with the approved guidelines and approved by the Research Council and Animal Care and Use Committee of the Third Military Medical University, Chongqing, China. Healthy 3 weeks old (~100 g) and adult (220–250 g) male Sprague-Dawley rats were acquired from the Animal Center of Xiaoqiao Hospital, Third Military Medical University.

### Ultrasonic therapeutic apparatus and microbubbles

The ultrasonic therapeutic apparatus and lipid-coated microbubbles with a mean particle diameter of 2 mm and at a concentration of 9 × 10^10^/mL (Department of Ultrasound, Xinqiao Hospital, Third Military Medical University, Chongqing, China) were used as reported in a previous study^11^, the results of that work showed that the combined use of ultrasound and microbubbles increased the prostate permeability. The ultrasound parameters were as follows: frequency: 1 MHz, acoustic pressure: 500 kPa, acoustic intensity: 0.023 W/cm^2^, duty cycle: 1.0%, and irradiation time: 5 minutes.

### Preparation of BMMSCs

Following the methods of Han *et al.*^19^, we successfully isolated, identified, and cultured BMMSCs. In a sterile environment, 3-week-old male Sprague-Dawley rats were euthanized and their bilateral femurs were isolated and repeatedly aspirated with PBS to collect the bone marrow suspension, which was centrifuged at 1000 r/min for 5 minutes to obtain a single cell suspension. Next, the cells were cultured to the third passage (P_3_). Then, anti-mouse CD29, CD44, and CD45 monoclonal antibodies were added to the P_3_ cells and positive labeling was identified using flow cytometry (Macrost, USA). The BMMSCs were then transfected with an adenovirus containing GFP and luc genes, and fluorescence microscopy was used to determine the transfection ratios.

### Study design and creation of the CBP model

Following the methods of Elkahwaji *et al.*^20^, the CBP model was established in the healthy, adult male Sprague-Dawley rats through ultrasound-guided prostate puncture and injection of *E. coli*. After 4 weeks, the model was established successfully, and the CBP rats were randomly divided into four groups: the CBP group, BMMSCs group, ultrasound group, and microbubble-mediated ultrasound group (MMUS group). A healthy control group was also prepared.

### Experimental treatments

The rats in the MMUS group were injected with microbubbles (0.1 mL/kg) and their prostates were insonated using the therapeutic apparatus. Next, BMMSCs (1 × 10^7^ BMMSCs in 300 μL of PBS) were injected into the rats via the caudal veins. In the ultrasound group, the prostates were insonated and then BMMSCs (1 × 10^7^ BMMSCs in 300 μL of PBS) were injected. In the BMMSCs group, the prostates received sham ultrasound exposure and then BMMSCs (1 × 10^7^ BMMSCs in 300 μL of PBS) were injected. In the CBP and healthy control groups, the prostates were treated with sham ultrasound exposure and an injection of an equivalent volume of saline.

### *In vivo* imaging

Twenty-four hours after the microbubble and ultrasound exposure, 3 rats from each of the BMMSCs, ultrasound, and MMUS groups were given an intraperitoneal injection of luciferase substrates. After another 24 hours, the distribution and amount of BMMSCs were detected using an *in vivo* imaging system.

### Histology

After 2 weeks, three rats per group were euthanized for histology. The prostates were incubated in 4% paraformaldehyde for 24 hours and then dehydrated in 85% ethanol for 30 min, 2 changes of 95% ethanol for 30 min, 3 changes of anhydrous ethanol for 30 min, acetone for 20 min, and 2 changes of xylene for 20 min. Next, the prostates were embedded in paraffin and cut into 5 μm tissue sections. These sections were stained with hematoxylin and eosin (HE) and then observed using light microscopy (BX50, Olympus, Tokyo, Japan).

### Enzyme-linked immunosorbent assay

Two weeks after the experimental treatment, 10 rats per group were euthanized in order to quantify the expressions of TNF-α and IL-1β in the prostate tissues using ELISA. The prostate tissues were manually minced and subsequently homogenized in a glass homogenizer at 4 °C. The contents of TNF-α and IL-1β were quantified by ELISA following the manufacturer’s instructions. The enzyme-labeled substrate was detected for analyses of the spectrophotometric values on a multimode reader (Thermo Fisher Scientific, Waltham, MA, USA).

### Real-time quantitative RT-PCR

Two weeks after the experimental treatments, three rats per group were euthanized in order to determine the mRNA expressions of TNF-α and IL-1β. For each rat, the entire prostate was manually minced and subsequently homogenized at 4 °C. The total RNA was extracted from the prostate tissue using TriZol (Invitrogen, Thermo Fisher Scientific), and cDNA was synthesized from 1 μg of total RNA from each sample using a standard reverse transcriptase reaction kit. The primers used for TNF-α were forward: 5′-CCCCGACTACGTGCTCCTC-3′ and reverse: 5′-GAA CGGATGAACACGCCAGTC-3′; for IL-1β were forward: 5′-CAAGGA GAGACAAGCAACGACAA-3′, and reverse: 5′-GTCCCGACCATTGC TGTTTC-3′; and for β-actin were forward: 5′-ACCCCGTGCTGCTGA CCGAG-3′, and reverse: 5′-TCCCGGCCAGCCAGGTCCA-3′. A total of 30 PCR cycles were used for amplification of TNF-α, IL-1β, and β-actin. We used the comparative CT method to estimate the relative quantification of TNF-α and IL-1β mRNA across multiple samples using the equation: R = 2^−ΔΔCT^, in which C_T_ is the cycle threshold for the gene as determined from the real-time PCR amplification plot and R is the relative quantification. The β-actin amplification levels were used as internal controls to normalize each sample’s C_T_ value as follows: ΔC_T_ = C_T β-actin_ − C_T TNF-α_ or C_T IL-1β_.

### Immunohistochemistry

After 2 weeks, the prostates from 4 rats per group were harvested and immediately frozen in liquid nitrogen to analyze the expression of TNF-α and IL-1β. The frozen prostates were cut into 5 μm sections, air-dried, and fixed in 4% paraformaldehyde for 15 min at room temperature. They were then incubated with 3% H_2_O_2_ in methanol for 30 min at room temperature and then washed and blocked with normal goat serum for 30 min. Next, the sections were incubated with rabbit polyclonal anti-TNF-α and anti-IL-1β antibodies (diluted 1:100 in PBS) at 4 °C overnight. Negative control sections were prepared by replacing the primary antibodies with PBS. To determine the expressions and distributions of TNF-α and IL-1β, the sections were imaged and analyzed with ImagePro-Plus software(Media Cybernetics, USA).

### Statistical analysis

All data are expressed as the mean ± SD, and Student’s t-tests were used to determine whether difference between groups were significant. One-way analysis of variance tests were used to determine the significance of differences among multiple groups. P-values less than 0.05 were considered statistically significant.

## Results

### Distribution and amount of BMMSCs after treatment

**Figure 1:**
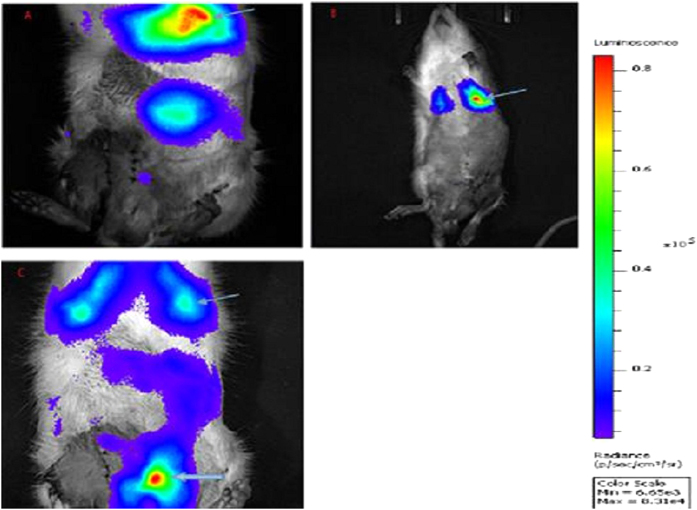
Distribution and amount of fluorescein-labeled BMMSCs in the different groups assessed by *in vivo* imaging. (**a**) In the ultrasound group, the fluorescein-labeled BMSCs were mainly found in the lung tissue (thin arrow) and epigastrium. (**b**) In the BMMSCs group, the fluorescein-labeled BMMSCs were found in the lung tissue (thin arrow). (**c**) In the MMUS group, more of the fluorescein-labeled BMMSCs were found in the prostate tissue than in the other groups (thick arrow).

In the BMMSCs and ultrasound groups, the fluorescein-labeled BMMSCs were mostly found in the lung, with a weak fluorescein signal found in the prostate tissue (Fig. 1a,b). In contrast, in the MMUS group, the fluorescein-labeled BMMSCs were found in the prostate and lung, and the fluorescent signal from the BMMSCs in the prostate tissue was much higher than that in the other groups (Fig. 1c).

### Histology

**Figure 2:**
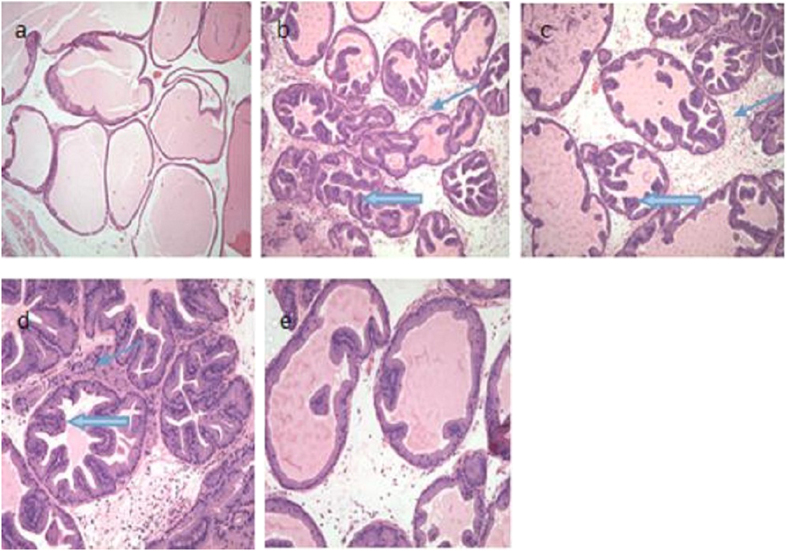
HE staining of prostate tissue from rats in the different groups (magnification: ×200). (**a**) The HE staining in the healthy control group showed no inflammatory cell infiltration or fibrous tissue hyperplasia with the pseudostratified prostate gland epithelial cells and glandular epithelial tissue retaining their regular arrangement. No glandular hyperplasia was found in the duct cavity. In the (**b**) CBP, (**c**) ultrasound, and (**d**) BMMSCs groups, a large number of infiltrated inflammatory cells (thin arrows) and fibrous tissue hyperplasia of the intercellular structure was found, as well as disorder in the prostate gland epithelial cell layer. A large number of tumor-like epithelial proliferations protruded into the duct cavity (thick arrow). (**e**) In the MMUS group, there was less inflammatory cell infiltration and fibrous tissue hyperplasia than with the other treatments, the amount of tumor-like epithelial proliferation in the duct cavity was clearly decreased, and the prostate glandular epithelium was arranged regularly.

The healthy control group showed no inflammatory cell infiltration or fibrous tissue hyperplasia, and the pseudostratified prostate gland epithelial cells and glandular epithelial tissue retained their normal arrangements. The nucleus and cytoplasm were uniform, and no tumor-like glandular hyperplasia was found in the duct cavity (Fig. 2a). In contrast, the prostate tissues from the CBP, BMMSCs, and ultrasound groups showed a large number of infiltrated inflammatory cells and fibrous tissue hyperplasia in the intercellular structure, as well as disorder in the prostate gland epithelial cell layers. The HE staining showed necrotic epithelial cells and a large amount of tumor-like epithelial proliferation protruding into the duct cavity (Fig. 2b–d). In the MMUS group, less inflammatory cell infiltration and fibrous tissue hyperplasia was found in the intercellular structures than that in the CBP, BMMSCs, and ultrasound groups. Similarly, the tumor-like epithelial proliferation in the duct cavity was clearly decreased, and the prostate glandular epithelium was arranged more regularly (Fig. 2e).

### ELISA quantification of TNF-α and IL-1β protein

As shown in Table 1, the amounts of TNF-α and IL-1β proteins in the CBP, ultrasound, and BMMSCs groups were all similar (P < 0.05) and significantly higher than that in the healthy control group (P < 0.05). In contrast, the amounts of TNF-α and IL-1β protein in the MMUS group (microbubble combined with ultrasound and BMMSCs) were significantly lower than those in the CBP, ultrasound, and BMMSCs groups (P < 0.05). In addition, the values were not different than those in the healthy control group (P > 0.05).

### mRNA expressions of TNF-α and IL-1β in prostate tissue

The real-time quantitative RT-PCR analysis showed the mRNA expression levels of TNF-α and IL-1β in each group (Tables 2 and 3). There were significant differences in the relative amounts of TNF-α and IL-1β expressions between the MMUS group and the CBP, ultrasound, and BMMSCs groups (P < 0.05). However, there was no difference between the MMUS group and the healthy control group (P > 0.05).

### Immunohistochemistry of TNF-α and IL-1β in prostate tissue

**Figure 3:**
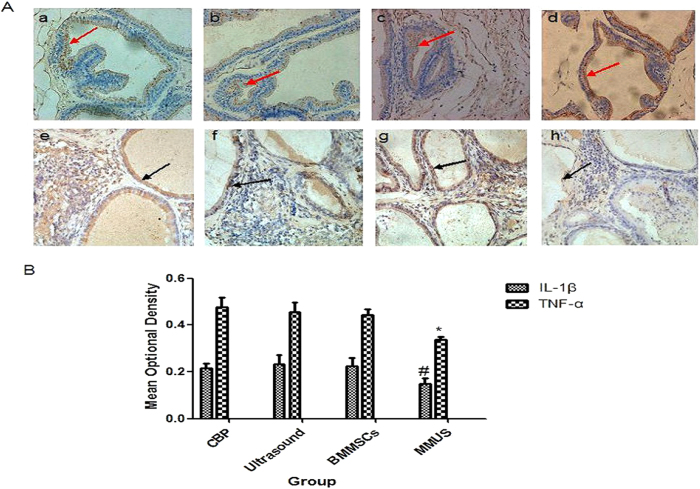
Presence and distribution of TNF-α and IL-1β in the prostate tissue were analyzed by immunohistochemical staining (scale bar: 20 μm). Representative images of TNF-α staining in the (**a**) CBP, (**b**) ultrasound, (**c**) BMMSCs, and (**d**) MMUS groups. Red arrows highlight TNF-α staining. Representative images of IL-1β staining in the (**e**) CBP, (**f**) ultrasound, (**g**) BMMSCs, and (**h**) MMUS groups. Black arrows highlight IL-1β staining. i) Mean optical densities (mean ± SD) of the TNF-α and IL-1β staining from four rats per group. * and ^#^P<0.05 vs. the CBP, ultrasound, and BMMSCs groups.

The immunohistochemistry showed the amounts and distribution of TNF-α and IL-1β after treatment. TNF-α and IL-1β were mostly secreted by the prostate gland epithelial cell layer and intercellular structure, with a small amount also found in the duct cavity (Fig. 3A). After treatment with microbubbles and ultrasound combined with BMMSCs, the staining for TNF-α and IL-1β was decreased compared to that found in the CBP, ultrasound, and BMMSCs groups (Fig. 3A(d,h)). The mean optical density values of the TNF-α and IL-1β staining are shown in Fig. 3B, indicating that there was no difference in their amounts between the CBP, ultrasound, and BMMSCs groups.

## Discussion

This study showed that BMMSCs entered the prostate after the prostate permeability was increased by microbubble-mediated ultrasound irradiation, which reduced the CBP-induced inflammation and decreased the expressions of TNF-α and IL-1β. These results suggest that microbubble-mediated ultrasound-induced prostate accumulation of BMMSCs may be an effective therapeutic method for CBP.

BMMSCs are rapidly proliferating, low immunogenic, pluripotent, and fibroblast- like adult stem cells^21^. In addition to being able to differentiate into various types of cells, BMMSCs also play an important role in the treatment of certain diseases. One study showed that the transplantation of BMMSCs significantly attenuated lung inflammation and other pathological changes in emphysemic rats^21^. BMMSCs have also been shown to have immunomodulatory capabilities caused by their secretion of several different growth factors^22^. In *E. coli*-induced pneumonia in rats, treatment with BMMSCs enhanced the phagocytosis of monocytes through secretion of keratinocyte growth factor and simultaneous secretion of the antibacterial peptides LL-37 and apolipoprotein-2 that directly eliminate *E. coli*^23^. In another study, allogeneic BMMSCs were transplanted into the gastrointestinal tract of rats, and they protected against ischemia/reperfusion injury by producing paracrine growth factors and anti-inflammatory cytokines and repairing the injured tissues^24^.

Inflammatory cytokines, which function as cell-to-cell signaling molecules, are produced by endothelial, epithelial, and other nonimmune cells and have a variety of crucial roles in regulating the immune response and mediating the inflammatory response^25^. The mediator role of cytokines in the pathogenesis of CBP has recently been given more attention. Cytokines act locally over short cellular distances as initiators and modulators of immune-induced inflammation^26^. One type of cytokine, TNF-α, is secreted by monocytes and macrophages and plays a prominent role in infection and inflammation. TNF-a may also be important for the host defense by exerting an anti-viral effect^27^. Many studies have reported that the TNF-α levels in seminal plasma are increased in CBP patients^28^. TNF-α acts on the prostate endothelial cells under bacterial stimulation to increase their expression of adhesion molecules, which leads to the local accumulation and infiltration of inflammatory cells. In addition, TNF-α also stimulates macrophages to secret more inflammatory mediators, such as prostaglandin, IL-6, and IL-1^29^. IL-1β, another type of cytokine, stimulates lymphocytes and monocytes to invade inflamed regions and induces a variety of inflammatory mediators, activating the inflammatory cascade^30^. During the pathogenesis of CP, IL-1β is a proximal cytokine and may play a role in the development of prostatitis because of its importance for inflammation and tissue repair^31^. IL-1β mainly stimulates the prostate gland epithelial cells to produce adhesion molecules and activates leukocytes to release proteolytic enzymes and oxygen free radicals. Simultaneously, it promotes the migration of T cells and monocytes and their accumulation in the prostate tissue, further resulting in an influx of white blood cells into the inflamed prostate. In a previous study, we found that antibiotics only inhibited the growth of bacteria in the prostate tissues, but did not prevent the development of inflammation or decrease the levels of inflammatory cytokines^12^. In the present study, we found that the inflammatory cell infiltration was significantly decreased after microbubble-mediated ultrasound-induced accumulation of BMMSCs and the expressions of TNF-α and IL-1β were also decreased. These results suggested that BMMSCs may be useful for treating CBP by their immunomodulatory regulation of the expressions of TNF-α and IL-1β.

In the male reproductive system, the blood–testis barrier and the blood–epididymal barrier protect the sperm against the interference and influence of external physiological and pathological factors. Are there other similar barrier structures in the male urinary and reproductive system? One study reported that damage to sperm occurred during the process of sperm and prostatic fluid mixing in the autoimmune rat model^32^. Another study showed that the concentrations of drugs in the prostatic secretion and prostate tissue are significantly lower than those found in the blood plasma^33^. These results indicated that a barrier structure, similar to the blood–testis barrier, existed between the blood and prostate duct cavity, and the functions of this barrier were to prevent inflammation and the migration of inflammatory cells and protect sperm from damage. Furthermore, hyperplasia of the fibrous tissue, thickening of the basement membrane, and compaction of the tight junctions between cells were increased in inflamed tissues. These pathological changes further strengthen the barrier functions^7^, which makes increasing the prostate permeability become a difficult challenge.

Microbubbles, which are a novel ultrasound contrast agent, not only enhance ultrasonography, but also mediate low-frequency ultrasonic insonation to increase tissue permeability, allowing drugs to attain higher concentrations in local tissue^34^. Many studies have reported that low-frequency ultrasound combined with microbubbles increase the permeability of the blood–tumor barrier^35^,^36^. Based on that work, our research group hypothesized that ultrasonic sonoporation increased prostate permeability^7^. In another study, we found that the concentrations of Evans blue in the prostate were increased after microbubble-mediated ultrasonic insonation, which indicated that microbubble-mediated ultrasonic insonation enhanced the permeability of the prostate and suggested a new method for increasing the effective concentrations of drugs^11^. In the present study, we did not find mitigation of the prostate tissue inflammation after treatment in the BMMSCs and ultrasound groups, and only a small amount of fluorescein-labeled BMMSCs accumulated in the prostate tissues of those groups. These results suggest that microbubble-mediated ultrasonic insonation increases the permeability of the prostate and that this method may be an effective therapeutic method for treating CPB and other prostate diseases.

Although our study showed a potential therapeutic effect of microbubble-mediated ultrasound-induced accumulation of BMMSCs on CBP, we did not observed whether there were any damages to the prostate tissues by using this method, hence, more studies need to be evaluated in the future.

## Conclusions

In summary, the present study demonstrates that microbubble-mediated ultrasound-induced accumulation of BMMSCs can inhibit the inflammatory response and decrease the expressions of TNF-α and IL-1β in prostate tissue of the CBP rat model. Based on these results, microbubble-mediated ultrasound-induced accumulation of BMMSCs may be an effective therapeutic method for treating CBP.

## Additional Information

**How to cite this article**: Yi, S. *et al.* Microbubble-mediated ultrasound promotes accumulation of bone marrow mesenchymal stem cell to the prostate for treating chronic bacterial prostatitis in rats. *Sci. Rep.* **6**, 19745; doi: 10.1038/srep19745 (2016).

## Figures and Tables

**Table 1:** Protein expressions of TNF-α and IL-1β in prostate tissue (mean ± SD).

	Healthy control	CBP	Ultrasound	BMMSCs	MMUS
TNF-α	16.78 ± 3.33	76.23 ± 13.78*	66.28 ± 8.93*	66.91 ± 6.57*	22.81 ± 8.16#
IL-1β	55.50 ± 9.40	326.10 ± 30.46^*^	321.00 ± 29.04^*^	300.45 ± 22.15*	56.09 ± 10.71#

^*^P < 0.05 vs. the healthy control group

^#^P < 0.05 vs. the CBP, ultrasound, and BMMSCs groups

No difference was found between the MMUS and healthy control groups (P > 0.05).

**Table 2:** Real-time quantitative RT-PCR analysis of the relative mRNA expressions of TNF-α in prostate tissue (mean ± SD).

Groups	C_T TNF-α_	ΔC_T_	ΔΔC_T_	2^−ΔΔCT^	C_T β-actin_
Healthy control	29.15 ± 0.02	3.46 ± 0.07	0	1.00 ± 0.05	25.70 ± 0.05
CBP	26.80 ± 0.06	1.03 ± 0.13	−2.43 ± 0.13	5.38 ± 0.47	25.76 ± 0.11
Ultrasound	26.48 ± 0.10	1.07±0.16	−2.40 ± 0.16	5.25 ± 0.58	25.48 ± 0.03
BMMSCs	27.91 ± 0.03	1.00 ± 0.18	−2.46 ± 0.18	5.53 ± 0.66	25.53 ± 0.04
MMUS	28.48 ± 0.03	3.03 ± 0.09	−0.43 ± 0.09	1.34 ± 0.08*#	25.60 ± 0.13

^*^P < 0.05 vs. the CBP, ultrasound, and BMMSCs groups

^#^P > 0.05 vs. the healthy control group.

**Table 3:** Real-time quantitative RT-PCR analysis of the relative mRNA expressions of IL-1β in prostate tissue (mean ± SD).

Groups	C_T TNF-α_	ΔC_T_	ΔΔC_T_	2^−ΔΔCT^	C_T β-actin_
Healthy control	31.07 ± 0.05	5.37 ± 0.03	0	1.00 ± 0.02	25.70 ± 0.05
CBP	28.67 ± 0.04	2.91 ± 0.13	−2.46 ± 0.13	5.41 ± 0.31	25.76 ± 0.11
Ultrasound	28.57 ± 0.05	3.09 ± 0.75	−2.28 ± 0.08	5.10 ± 0.231	25.48 ± 0.03
BMMSCs	29.84 ± 0.03	4.31 ± 0.06	−1.06 ± 0.06	4.93 ± 0.37	25.53 ± 0.04
MMUS	30.40 ± 0.07	4.87 ± 0.05	−0.50 ± 0.05	1.42 ± 0.49*#	25.60 ± 0.13

^*^P < 0.05 vs. the CBP, ultrasound, and BMMSCs groups

^#^P > 0.05 vs. the healthy control group.
